# Comprehensive protein tyrosine phosphatase mRNA profiling identifies new regulators in the progression of glioma

**DOI:** 10.1186/s40478-016-0372-x

**Published:** 2016-09-01

**Authors:** Annika M. Bourgonje, Kiek Verrijp, Jan T. G. Schepens, Anna C. Navis, Jolanda A. F. Piepers, Chantal B. C. Palmen, Monique van den Eijnden, Rob Hooft van Huijsduijnen, Pieter Wesseling, William P. J. Leenders, Wiljan J. A. J. Hendriks

**Affiliations:** 1 Department of Cell Biology, Radboud Institute for Molecular Life Sciences, Radboud University Medical Center, Geert Grooteplein 26, 6525 GA Nijmegen, The Netherlands; 2 Department of Pathology, Radboud Institute for Molecular Life Sciences, Radboud University Medical Center, Nijmegen, The Netherlands; 3 Department of Pathology, VU University Medical Center, Amsterdam, The Netherlands; 4 Merck Serono S.A, Geneva, Switzerland

**Keywords:** Glioblastoma, Astrocytoma, EGFR, Oligodendroglioma, IDH1, DUSP26, MTMR4, PTEN, PTP, PTPRM, PTPRN2, PTPRT, PTPRZ1, Malignancy

## Abstract

The infiltrative behavior of diffuse gliomas severely reduces therapeutic potential of surgical resection and radiotherapy, and urges for the identification of new drug-targets affecting glioma growth and migration. To address the potential role of protein tyrosine phosphatases (PTPs), we performed mRNA expression profiling for 91 of the 109 known human PTP genes on a series of clinical diffuse glioma samples of different grades and compared our findings with in silico knowledge from REMBRANDT and TCGA databases. Overall PTP family expression levels appeared independent of characteristic genetic aberrations associated with lower grade or high grade gliomas. Notably, seven PTP genes (*DUSP26*, *MTMR4*, *PTEN*, *PTPRM*, *PTPRN2*, *PTPRT* and *PTPRZ1*) were differentially expressed between grade II-III gliomas and (grade IV) glioblastomas. For *DUSP26*, *PTEN*, *PTPRM* and *PTPRT*, lower expression levels correlated with poor prognosis, and overexpression of *DUSP26* or *PTPRT* in E98 glioblastoma cells reduced tumorigenicity. Our study represents the first in-depth analysis of PTP family expression in diffuse glioma subtypes and warrants further investigations into PTP-dependent signaling events as new entry points for improved therapy.

**Electronic supplementary material:**

The online version of this article (doi:10.1186/s40478-016-0372-x) contains supplementary material, which is available to authorized users.

## Introduction

Gliomas arise from glial (precursor) cells and represent the most frequent type of primary brain tumor. The vast majority is classified as diffuse gliomas, reflecting their infiltrative growth into the brain parenchyma along myelinated axon bundles and blood vessels [1, 2]. Diffuse gliomas are histopathologically graded into WHO grades II-IV, with grade IV astrocytoma (glioblastoma) being most malignant [3]. Their diffuse infiltrative character severely complicates glioma treatment. Whereas surgery and radiotherapy mostly address the central part of the tumor, cells that have migrated into the surrounding brain tissue remain relatively unaffected and give rise to recurrences. Although glioblastoma patient survival time has slightly improved over the past decades, the prospect with current treatment is only a median 15 months following diagnosis [4], urging for the development of novel drug modalities to combat these tumors.

Diffuse gliomas can also be subdivided into several classes based on molecular criteria. Lower grade (grade II-III) gliomas and secondary grade IV glioblastomas (arising from lower grade gliomas) display mutations in isocitrate dehydrogenase genes (*IDH1*/*2*) in 80 % of the cases [5]. Primary glioblastomas (originating *de novo*) are wild type for *IDH1* and *IDH2* [6]. Presence of these mutations is now often used as an independent predictor of prolonged survival [7, 8]. Glioblastomas have been further subdivided on the basis of transcriptional profiles as cancers of the pro-neural, neural, mesenchymal and classical subtype [9, 10]. Pro-neural and neural glioblastomas display *IDH1*/*2* mutations, whereas classical and mesenchymal gliomas often show loss of chromosome 10 (containing the tumor suppressor gene *PTEN* at 10q23), and/or amplification of chromosome 7 (containing the proto-oncogene *EGFR* at 7p12) [3]. Constitutively active mutant versions of receptor tyrosine kinases (RTKs), *e.g*. EGFR (EGFRvIII), PDGFRA or MET (METΔ7–8 [11]), are commonly encountered in primary glioblastomas [12]. As a result, in about 90 % of primary glioblastomas the RTK/RAS/PI3K signaling pathway is affected by mutations [10], underscoring deregulated phosphotyrosine-based signaling as a major driver in glioma etiology.

Protein tyrosine phosphorylation is controlled via balanced actions of protein tyrosine kinases and protein tyrosine phosphatases (PTPs) [13]. Tumor cell biological research has mostly focused on the druggable kinases, and many tyrosine kinase genes are listed as proto-oncogenes (*e.g. EGFR*, *MET*, *ERBB2*, and *PDGFRA*) [10]. Consequently, investigations on the counteracting, hence potentially tumor suppressive, PTPs [13, 14] lag behind. A well-known exception is PTEN [15, 16], the PTP family member that is mutated or deleted in 41 % of primary glioblastomas [10] and whose tumor suppressive action is linked to its phospholipid phosphatase activity [17]. Although seemingly counter-intuitive, it has become clear that some PTPs (e.g. SHP2, DUSP6 and PTPRZ1) behave as oncogenes [18, 19]. Thus, the relevance of specific PTPs in certain cancer types is emerging [19, 20] but PTPome studies in diffuse glioma specimens are lacking so far.

We performed expression profiling of the PTP gene family in diffuse glioma samples of different grades and genetic backgrounds, and found that overall PTP expression levels decrease with increasing glioma grade and appear independent of characteristic genetic aberrations. Furthermore, for two of the PTPs whose expression levels differed consistently and significantly between lower grade (II-III) and high grade (IV, glioblastoma) gliomas and correlated with patient survival, we could demonstrate tumor suppressive activity. These findings highlight PTP impact on glioma tumorigenicity.

## Materials and methods

### Tumor samples and cell lines

Analyses of patient material were performed in accordance with the guidelines of the local medical ethical committee of the Radboud University Medical Center, Nijmegen, The Netherlands. Patient glioma samples (*n* = 83), comprising 19 grade II, 15 grade III and 49 grade IV tumors, were obtained from the archives of the Radboud University Medical Center [see Additional file 1]. Histologically normal brain control tissue (temporal neocortex and white matter) was obtained from surgery on epilepsy patients at the VU University Medical Center, Amsterdam, The Netherlands.

HEK293FT cells were purchased from Invitrogen, LN-229 glioblastoma cells were from the American Type Culture Collection, and U-251 MG cells were kindly provided by Joost Schalkwijk (Radboudumc). Derivation and use of the xenograft-derived glioblastoma cell model E98 has been described elsewhere [21]. U-251 MG, LN-229, HEK293FT and E98 cells were cultured in high glucose Dulbecco’s Modified Eagle Medium (Life Technologies; cat.no.11960–044) supplemented with 10 % Fetal Bovine Serum (PAA laboratories; Cat.no. A15–101), 4 mM glutamine and 1 mM pyruvate (Life Technologies), at 37 °C in a humidified incubator under 7.5 % CO_2_. For some experiments 1 μM Gefitinib (Selleck Chemicals), 4 mM 2-hydroxyglutarate (Sigma H8378), or DMSO (solvent control) was added 48 h prior to isolation.

### RNA isolation

Total RNA was isolated from frozen tumor material or from cell lines. Ten 4 μm cryosections were homogenized in 1.3 ml RNA-Bee (Tel-Test Inc., cs104B) through mechanical dissociation. Next, 260 μl chloroform was added and samples were vortexed and incubated on ice for 15 min before being centrifuged at maximum speed for 15 min at 4 °C. The top clear supernatant was transferred into a new tube and RNA was precipitated with 1 volume of isopropanol. The RNA pellet was dissolved in 400 μl NSE (50 mM NaAc, pH 5.2; 0.2 % SDS; 2 mM EDTA in RNase-free water) and re-precipitated with 1 ml ethanol and collected by centrifugation (30 min at 4 °C, 14,000 g). The RNA pellet was briefly air-dried and dissolved in 30 μl of RNase-free water. Concentrations were determined on a NanoVue plus spectrophotometer (GE Healthcare).

### RT-reaction

Reverse transcriptase reactions were performed using the iScript^tm^ cDNA synthesis kit (Bio-Rad). RNA concentrations that were used ranged from 20 ng/μl to 2 μg/μl, and applied RNA and RT-mix volumes were according to the manufacturers’ specifications. Synthesis of cDNA was performed by incubating for 5 min at 25 °C, followed by 30 min at 42 °C and 5 min at 85 °C. Resulting cDNA samples were diluted in distilled water (1:3 – 1:5) and stored at −80 °C until further use.

### qRT-PCR

Validated qPCR primer sets for mRNA transcripts from 91 PTPs and housekeeping genes *ACTB*, *CANX* and *PSMB* (described in [22]) were purchased from Qiagen and SABiosciences. Transcript abundance was determined real-time in 10 μl reactions using SYBR GREEN (Bio-Rad) and 3 μl of the diluted cDNA samples on a CFX96™ system using the C1000™ Thermal Cycler (Bio-Rad). PCR reactions were initialized at 95 °C for 15 min and followed by 40 cycles of 15 s at 95 °C and 40 s at 60 °C. To monitor primer specificity, at the end of the last cycle a melting curve with increments of 0.5 °C was recorded between 60 °C and 95 °C. PTP transcript amounts were normalized to housekeeping gene expression levels (ΔC_t_). Normalization to any of three housekeeping genes gave comparable results, and values normalized towards *ACTB* are used here. The ΔC_t_ values are presented relative to the transcript levels in control brain tissue, according to the ΔΔC_t_ method of Livak and Schmittgen [23]. The average expression level for a given PTP was considered to be meaningfully different between tumor grades when *p* < 0.05 and │ΔΔC_t_│ > 1.5.

### Tissue micro-array staining

Immunohistochemical stainings were performed on 4-μm sections of tissue micro-arrays (TMAs) containing formalin-fixed paraffin-embedded (FFPE) glioma (lower grade, *n* = 16; high grade, *n* = 47). Slides were deparaffinized and rehydrated according to standard methods. Heat-induced epitope retrieval (HIER) was performed in TRIS/EDTA buffer, pH 9 (Klinipath), for 10 min in a microwave oven at 180 W. After cooling down, endogenous peroxidase activity was blocked using 3 % H_2_O_2_ in PBS for 10 min at room temperature. After washing with PBS, primary antibodies against PTPRZ1 (BD Transduction Laboratories; #610179), DUSP26 (Atlas antibodies; HPA018221), MTMR4 (Pierce; #Pa5–13711), or PTPRT (Ab frontier; #LFMA0345 clone T20–3C7) were diluted in Normal Antibody Diluent (ImmunoLogic) and incubated for 1 h at room temperature. Slides were then washed with PBS and incubated with Poly-HRP-GAM/R/R IgG (ImmunoLogic) as secondary antibody for 30 min at room temperature prior to detection with 3,3-diaminobenzidine staining (Power-DAB, ImmunoLogic). All sections were counter-stained with haematoxylin and mounted in QuickD Mounting Medium (Klinipath). Sections were scored by two observers (AB and KV), after instructions and partly supervised by a neuropathologist (PW), for intensity and percentage of cells stained. Samples were binned into three classes: high staining (high intensity staining, in the majority of tumor cells), medium staining (lower intensity staining, in a considerable amount of tumor cells) and low or no staining (low intensity or no staining, in a minor part of the tumor cells). For DUSP26 we defined a sample as low staining if there were positive cells in the sample and as no staining if not a single stained tumor cell was present.

### Generation of cells with aberrant EGFR expression

Third generation lentiviral constructs that drive expression of wildtype (EGFR WT) or variant III (EGFRvIII) epidermal growth factor receptor were generated as follows. A NotI linker (self-annealed 5′- CTA GTC CGG GCG GCC GCC CGG A–3′) was ligated into the cleaved XbaI site in PRK5-EGFR-WT (a mammalian expression vector harboring wild-type human EGFR cDNA, kindly provided by Reiner Lammers, Tübingen, Germany). The resulting plasmid was digested using NotI and Hin*d*III and the EGFR WT-encoding fragment was ligated into NotI/Hin*d*III-digested pENTR/NotI-XhoI [21]. EGFRvIII cDNA was obtained using primers fw: 5′- GAT ATC ACC ATG CGA CCC TCC GGG–3′ and rev: 5′- CAG CGG CCG CTC ATG CTC CAA TAA ATT CAC TG–3′ in a PCR reaction with an EGFRvIII-positive glioblastoma cDNA pool as template. The amplicon was digested with NotI and resulting NotI/blunt-end EGFRvIII cDNA was ligated into Hin*c*II/NotI-digested vector pENTR-1A (Invitrogen). Resulting pENTR plasmids were subjected to an LR reaction (Gateway® LR clonase^tm^ II Enzyme mix; Invitrogen # 11791–020) with pLenti6.2/V5-DEST as destination vector, and pENTR/NotI-XhoI served to generate the empty vector control.

Lentiviruses were generated in HEK293FT cells as described earlier [21]. Small samples of virus-containing medium were diluted 1:2 with methanol to monitor viral titers by dot blot on nitrocellulose membranes (0.45 μM Schleicher&Schuell #401196). After blocking for 30 min with 3 % non-fat dry milk in TBST (10 mM Tris–HCl, pH 8.0; 150 mM NaCl; 0.1 % Tween-20 (Sigma-Aldrich)) membranes were incubated with anti-VSV monoclonal antibody P5D4 [24] in blocking buffer for 1 h. Membranes were then washed three times for 10 min in TBST and subsequently incubated with IRDye® 800CW-conjugated goat-anti-mouse IgG (Li-COR Biosciences, #926–32350) for 1 h at room temperature in the dark. Blots were analyzed using the Odyssey imaging system (LI-COR Biosciences).

U-251 MG glioblastoma cells were transduced with lentiviral expression constructs for EGFR WT, EGFRvIII or empty vector control, and cultured in blasticidin-containing medium for 2 weeks. Resulting cell pools were cultured for 48 h in presence or absence of Gefitinib before being harvested for RNA and protein isolation. RNA was used for qRT-PCR as described above, to quantify transcript levels of three genes; *PTPRT*, *DUSP16* and *PTPRG*. Proteins from cell lysates were size-separated on 8 % SDS-PAGE gels and immunoblotted as described previously [21] except that for detection of phosphorylation-dependent epitopes 5 % Bovine Serum Albumin in TBST was used as blocking and incubation buffer. Primary antibodies were directed against EGFR (rabbit 1:2000 SC03; #1005), pTyr (mouse 1:1000 pY20; sc508), and GAPDH (rabbit 1:5000; Cell Signaling Technology #2118). Secondary antibodies were IRDye-680- or −800-conjugated goat-anti-mouse and goat-anti-rabbit antibodies. Detection was performed on the Odyssey imaging system.

### Generation of glioblastoma cells with PTEN deletion

PTEN-deficient LN-229 cells were created using CRISPR/Cas9 technology and a previously described PTEN targeting sequence [25] aimed at the start of the coding sequence in exon 1 of *PTEN*. Oligonucleotides that contained the target sequence and overlapped with sequences in the receiving guide RNA expression plasmid (PTEN-CRISPR-1-Fw: 5′- TTT CTT GGC TTT ATA TAT CTT GTG GAA AGG ACG AAA CAC CGG ATC GTT AGC AGA AAC AAA -3′; PTEN-CRISPR-1-Rev: 5′- GAC TAG CCT TAT TTT AAC TTG CTA TTT CTA GCT CTA AAA CTT TGT TTC TGC TAA CGA TCC -3′) were annealed and extended using Klenow DNA Polymerase fragment. The PTEN CRISPR-1 heteroduplex was introduced into the AflII-linearized gRNA cloning vector (a gift from George Church; Addgene plasmid # 41824 [26]) using Gibson assembly (New England Biolabs) according to manufacturer’s instructions. PTEN wildtype LN-229 glioma cells were transfected with a 1:1:0.1 mixture of the PTEN-targeting gRNA plasmid, plasmid hCas (a gift from George Church; Addgene plasmid # 41815 [26]), and a GFP expression plasmid (pLenti6/shSCR-GFP [21]), respectively, using jetPRIME (Polyplus; Cat.no 114–07). Individual clones were derived via limiting dilution and tested for successful CRISPR/Cas9-mediated PTEN gene editing using a T7 endonuclease I assay. Briefly, cells were lysed (100 mM Tris–HCl, pH 7.5; 5 mM EDTA; 0.2 % SDS; 200 mM NaCl; 100 μg/mL Proteinase K) overnight at 50 °C. Subsequently, samples were cleared by centrifugation (5 min, 8,000 rpm) and DNA in the supernatant was precipitated using an equal volume of isopropanol, washed once with 70 % ethanol, air-dried and finally dissolved in T_10_E_0.1_ (10 mM Tris–HCl, pH 8.0; 0.1 mM EDTA). Genomic DNA was then used in a PCR reaction with Q5 polymerase (NEB) containing Q5 GC enhancer according to manufacturer’s instructions. Primers used were: PTEN-T7-fw: 5′-GGC TGG GAA CGC CGG AGA GT-3′ and PTEN-T7-rev: 5′-CTA TCC TAC ATC ACC CCA TAG GG-3′. Amplicons were purified using the NucleoSpin® Gel and PCR Clean-up kit (Macherey-Nagel) and subjected to a T7 endonuclease I digestion assay (NEB, M0302S) for 15 min according to manufacturer’s instructions. Clones displaying edited PTEN alleles were further tested for PTEN status through qRT-PCR and immunoblot analyses as described above. Primary antibodies used were: rabbit anti-PTEN (1:2,000, CST#9559), mouse anti-tubulin monoclonal E7 (1:5,000; DSHB, University of Iowa), and rabbit anti-pAKT-Ser473 (1:2,000; CST #4058).

### PTPRT and DUSP26 expression constructs

PTPRT cDNA encompassing the full-length coding sequence was isolated from plasmid pGW-PTPRT (a kind gift from Jae-Ran Lee, Daejeon, Korea) using Bam*H*I and NotI, and ligated into the corresponding sites in vector pENTR/NotI-XhoI [21]. Similarly, plasmid pEF-HA-DUSP26 (a kind gift from Rafael Pulido, Barakaldo, Spain) was digested using Eco*R*I and XbaI and the DUSP26 open reading frame was ligated into pENTR/NotI-XhoI. An empty vector control (EV) was constructed as described above. An EGFP vector control was generated by PCR (Fw: 5′- TAT AGG ATC CAT GGT GAG CAA GGG CGA GG -3′; Rev: 5′- TAT ACT CGA GTT ACT TGT ACA GCT CGT CC -3′) using pEGFP as template, and the obtained amplicon was digested with Bam*H*I and XhoI and ligated into pENTR/NotI-XhoI. All resulting plasmids were used in LR Gateway® reactions (Invitrogen) using pLenti6/PGK-DEST-TagRFP [21] as destination vector. Obtained expression plasmids were used in HEK293FT cells to produce lentiviruses, as described above. E98 human glioblastoma cells [27] were lentivirally transduced twice, and 3 days later further processed for glioma cell behavior assays.

### Proliferation and migration assays

E98 cells were grown on collagen I-coated (10 μg/cm^2^; Invitrogen) coverslips to 60–80 % confluency over 48 h, and incubated for 1 h with culture medium containing 10 μM EdU (5-ethynyl-2′-deoxyuridine). EdU incorporation was visualized using the click-iT® EdU Imaging kit (Thermo Fisher Scientific, #C10086) via the manufacturers’ instruction. Coverslips were mounted on microscope slides in DAPI-containing Mowiol (Sigma-Aldrich) and images were collected on a Leica DMRA Fluorescence microscope, equipped with a DFC340 FX CCD camera, using 40x and 63x objectives. DAPI- and EdU-positive nuclei were counted automatically using FIJI software [28].

Migration of E98 cells was assessed in spheroid outgrowth assays as follows. E98 spheroids were generated in hanging drops using methylcellulose (12 mg/mL; Sigma, M6385) in DMEM supplemented with 10 % FCS (2500 cells per spheroid). The next day, individual spheroids were seeded in a 96-well imaging culture dish (BD Falcon, #353219) on top of a confluent mouse astrocyte layer in Matrigel-coated (30 μg/mL PBS; BD Biosciences, #356237) culture wells. 24 h later, cells were fixed and fluorescent (tagRFP) images were collected. Average migration distance of cells from spheroids (*n* > 37), calculated as change in radius of the spheroid over 24 h, were analyzed semi-automatically using FIJI software.

### In silico analyses

REpository for Molecular BRAin Neoplasia DaTa (REMBRANDT) was used to determine Kaplan-Meier curves based on PTP transcript levels (as determined via micro-array analyses) using the online Project Betastasis representation tool (http://betastasis.com/glioma/rembrandt/). Threshold was set at median expression for the respective PTP transcript. RNA-seq data from two different TCGA datasets (*Brain Lower Grade Glioma* [29] and *Glioblastoma Multiforme*; Provisional) were downloaded using cBioPortal (http://www.cbioportal.org/index.do) [30, 31]. These represented RNA-seq data of 166 *IDH1* WT glioblastoma samples, 9 *IDH1* mutant glioblastoma samples, 221 *IDH1* mutant lower grade glioma samples and 309 *IDH1* WT lower grade glioma samples.

### Statistics

Statistical significance was tested using non-paired two-tailed Student’s *t*-test or non-parametric Kruskal-Wallis one-way analysis of variance with post-hoc Dunn’s Multiple Comparison Test. Patient survival data was automatically calculated by Project Betastasis using a log-rank test. Heat map was generated in R (version 3.0.1).

## Results

### Differential expression of PTP transcripts between lower grade and high grade glioma samples

**Fig. 1:**
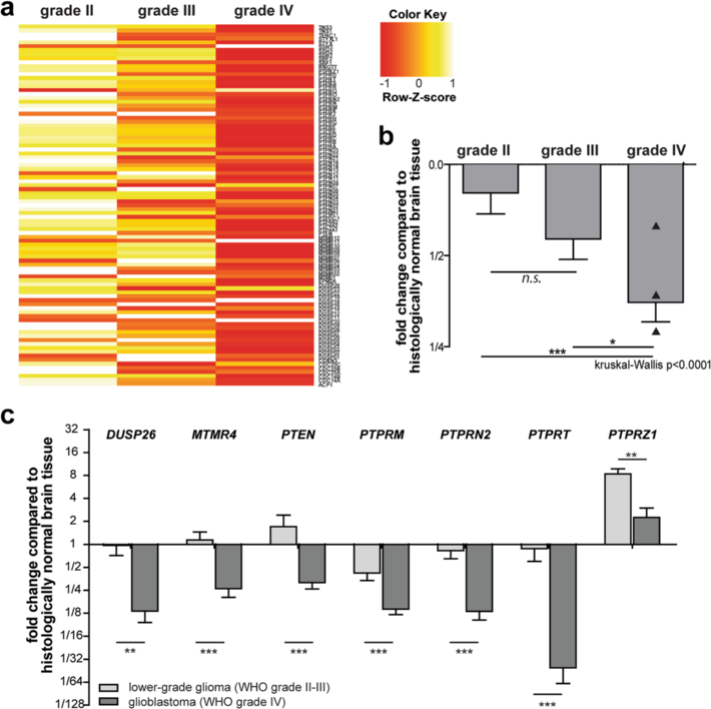
Seven PTP genes display significantly different expression levels in lower grade gliomas and glioblastomas. **a** Heat map of qPCR-based PTP expression levels – as determined in 19 WHO grade II, 15 WHO grade III and 49 WHO grade IV glioma samples – separated on malignancy grade and normalized per PTP as Z-scores. PTPs are represented in reverse alphabetical order ranging from TNS3 at the top to ACP1 at the bottom. **b** Overall PTP expression level difference between grade II, III or IV glioma samples and normal brain control tissue. The mean ΔΔC_t_ value over the 91 PTP primer sets in the grade II, III or IV samples is plotted. Bars indicate SEM; black triangles in grade IV bar represent secondary glioblastoma samples. Significance of mean expression differences between grades was calculated using Kruskal-Wallis one way analysis of variance with post-hoc Dunn’s Multiple Comparison Test (*n.s*. indicates non significant; * *p* < 0.05; ***p < 0.001). **c** Graph showing differential expression between lower grade (WHO grade II-III) and glioblastoma (WHO grade IV) glioma samples for seven PTPs on a ^2^log scale. PTP mRNA expression levels were determined by qRT-PCR, normalized to β-actin transcript levels, and depicted relative to the levels in histologically normal brain tissue. Values obtained in the first cohort of tumor samples are depicted [see Additional file 2]. Error bars indicate standard error of the mean (SEM). Asterisks indicate significance levels (** *p* < 0.01; *** *p* < 0.001) as determined by the two-tailed Student’s *t*-test

To investigate which members of the family of PTPs play roles in the biology of gliomas of different grades, we exploited a previously established and validated set of qPCR primers for 91 human PTP transcripts [22] to screen glioma specimens. Following cDNA synthesis and qPCR, PTP transcript levels were determined relative to those in control brain tissue. Strikingly, a pattern emerged showing significantly lower overall PTP transcript levels in the glioblastoma samples as compared to lower grade gliomas (Fig. 1a-b). In the first cohort of samples, expression levels of 36 PTPs differed significantly between lower grade (WHO grade II-III; *n* > 11) and glioblastoma (WHO grade IV; *n* > 15) samples [see Additional file 2]. To validate these findings, a second, independent cohort was tested with these 36 primer sets [see Additional file 3], resulting in the identification of seven PTP genes (*DUSP26*, *MTMR4*, *PTEN*, *PTPRM*, *PTPRN2*, *PTPRT* and *PTPRZ1*) that consistently and significantly differed in expression between lower and high grade gliomas (Fig. 1c).

**Fig. 2:**
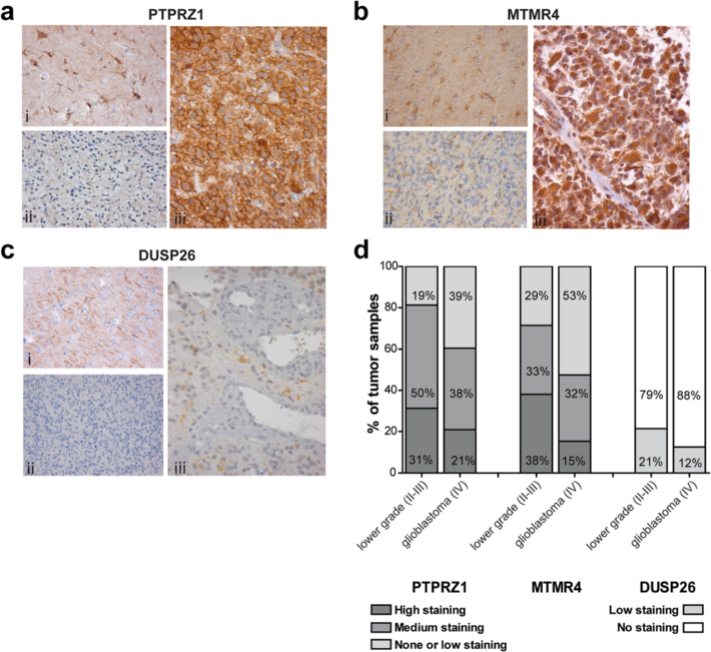
Immunohistochemical analysis of PTPRZ1, MTMR4 and DUSP26 expression in normal brain and glioma tissue. Representative images of formalin-fixed paraffin-embedded specimens stained with antisera against PTPRZ1 (**a**), MTMR4 (**b**) or DUSP26 (**c**). For PTPRZ1 and MTMR4 antisera, immunostainings in (i) normal brain tissue, (ii) negative or low intensity tumor areas and (iii) high intensity tumor areas are provided. For anti-DUSP26 a low staining example is shown in (iii). **d** Trichotome (PTPRZ1, MTMR4) and dichotome (DUSP26) analysis of PTP immunopositivity in lower grade and high grade specimens. Percentages reflect the proportion of samples in the respective bin

To investigate whether differences in transcript levels are reflected at the protein level, we performed immunohistochemical (IHC) staining on tissue micro-arrays containing 47 glioblastoma, 16 lower grade glioma samples and normal brain tissue. IHC could not be performed for *PTPRN2*-encoded IA-2β protein due to lack of appropriate antibodies. PTPRT protein levels were below detection limits in both normal brain tissue and in glioma tumor samples [see Additional file 4], in line with the high qPCR C_t_ values obtained for this PTP. Reduced PTEN and PTPRM protein levels in glioma specimens have been well documented (e.g. [19, 32]). The other PTPs were amenable for analysis by IHC. PTPRZ1 immunostainings revealed a clear membrane staining that was higher in lower grade specimens than in high grade counterparts (Fig. 2a), substantiating our RNA data and earlier reports [19]. Immunodetection of MTMR4 resulted in high signals in normal brain tissue in structures resembling astrocytes (Fig. 2b). In all samples (tumor and normal) also strong staining of the neuropil could be observed, with some samples additionally showing strong cytoplasmic staining in tumor cells (Fig. 2biii). DUSP26 was detected in neurons and neuropil of normal brain tissue but staining was usually low or absent in tumor areas (Fig. 2c). Low-level DUSP26 stainings were observed in 21 and 12 % of lower grade and glioblastoma samples, respectively (Fig. 2d), in concordance with our qPCR findings (Fig. 1c). Also MTMR4 expression levels were higher in grade II-III gliomas than in glioblastoma samples (Fig. 2d), in line with our RNA expression data (Fig. 1c).

### PTP expression patterns correlate with disease outcome

**Fig. 3:**
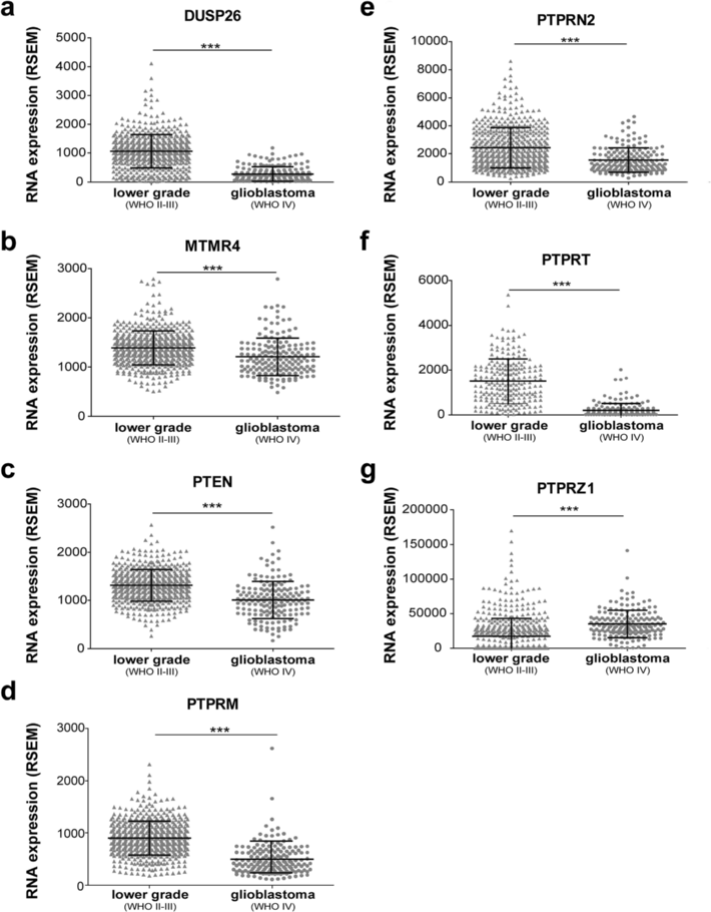
RNA-seq reveals PTP expression differences in lower grade glioma and glioblastoma. **a**-**g** Dot plots showing the mean estimated fraction of transcripts per million as calculated in RSEM (RNA-Seq by Expectation-Maximization; horizontal black bars) and standard deviation for the indicated PTP in lower grade glioma (WHO grades II-III; triangles) or glioblastoma (WHO grade IV; dots) tumor samples, as extracted from two TCGA RNA-seq datasets. Asterisks indicate significance levels (***p < 0.001) as determined by the two-tailed Student’s *t*-test

To corroborate our data, we performed in silico analysis of RNA-seq data from two TCGA glioma datasets and of micro-array data and survival probabilities as documented in the REMBRANDT brain tumor database. Out of the seven PTPs, all but PTPRZ1 had significantly lower expression levels in glioblastoma tumors as compared to lower grade glioma samples (Fig. 3), in agreement with our qPCR findings. Especially DUSP26 and PTPRT show a very strong difference between grades. PTPRZ1 is in fact overexpressed in both glioma subgroups as evident by the high RSEM RNA expression values.

**Fig. 4:**
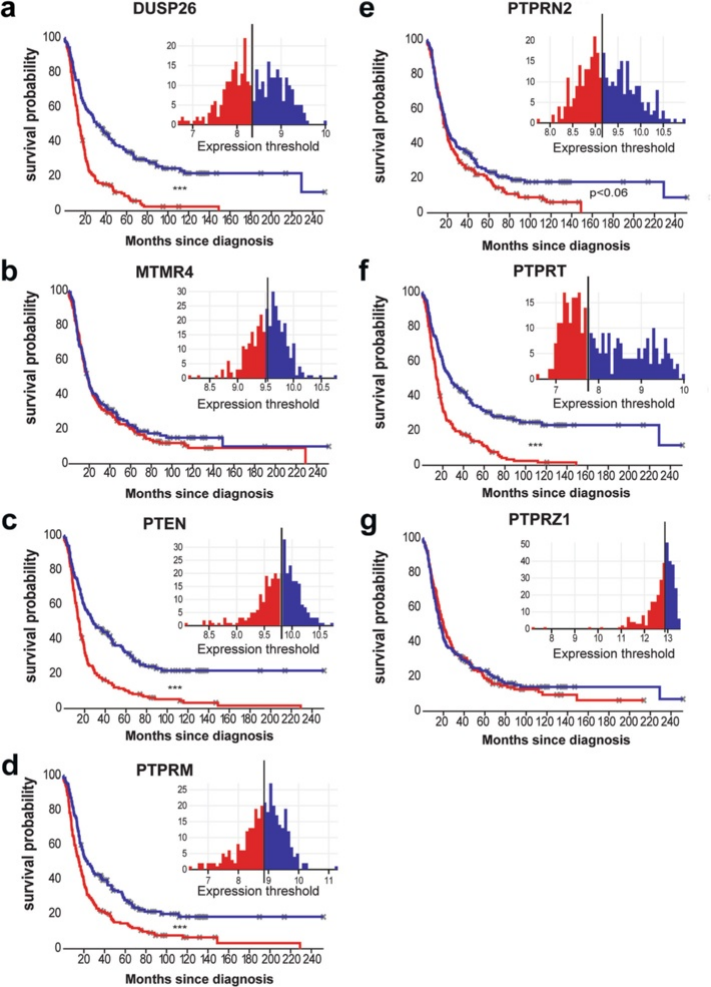
Transcript levels of several PTPs correlate with patient survival probability. **a**-**g** Kaplan-Meier analysis of glioma patient survival data according to low (red) and high (blue) PTP mRNA expression, obtained from microarray analyses on 524 glioma samples as present in the REMBRANDT repository. Insets show histograms of PTP expression distribution with the vertical black line indicating the cutoff point (taken at the median expression level) between low and high expressing samples. Asterisks indicate significance levels (***p < 0.001) calculated using the log-rank test in the Project Betastasis online representation tool

Using the Project Betastasis online representation tool, we subdivided all available tumors represented in the REMBRANDT dataset into low- and high-expressing types on the basis of available RNA micro-array data for our set of PTPs. Kaplan-Meier curves revealed a strong and significant correlation between low DUSP26, PTPRT, PTEN and PTPRM expression and disease progression (*p* < 0.001; Fig. 4a,f,c,d). MTMR4, PTPRN2 and PTPRZ1 did not correlate with survival (Fig. 4b,e,g). Also within the individual histological types (oligodendroglioma (grade II-III), astrocytoma (grade II-III) and glioblastoma (grade IV)) we observed expression-dependent survival probabilities for DUSP26 and PTPRT (data not shown). PTEN expression correlated with survival probability in glioblastoma and astrocytoma samples, while for PTPRM such an expression-dependent effect only holds for glioblastoma specimens (data not shown).

### Characteristic genetic aberrations in gliomas do not drive PTP expression

**Fig. 5:**
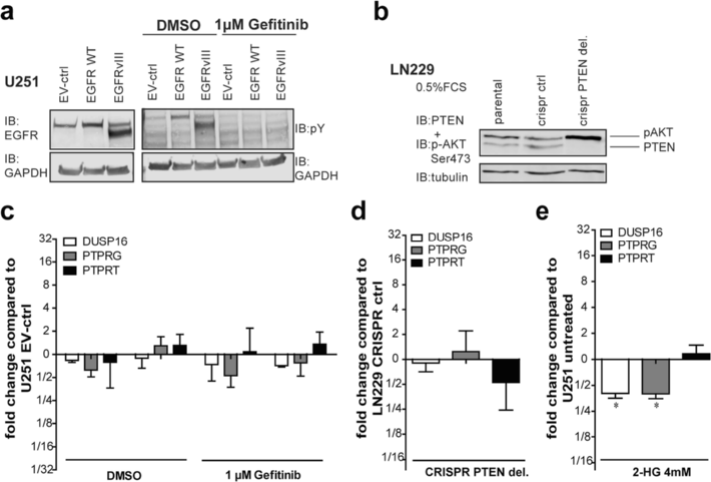
Characteristic mutations for lower/high grade gliomas exert no overt effect on PTP expression patterns. **a** Immunoblot of lysates from U-251 MG glioblastoma cells expressing wild-type EGFR, EGFRvIII or empty vector (EV) control. Cells were treated with the EGFR inhibitor Gefitinib (or DMSO as solvent control) for 48 h before being lysed. Immunostaining (IB) was with antibodies against EGFR, phosphotyrosine (pY) or GAPDH (as loading control). **b** Immunoblot analysis for PTEN and Ser473-phosphorylated AKT levels in parental and (PTEN-targeted) CRISPR/Cas9-treated LN-229 cells. Lysates were prepared following 8 h of serum starvation (0.5 % FCS), and tubulin immunostaining served as loading control. **c**-**e** Analysis of (*ACTB*-normalized) *DUSP16*, *PTPRG* and *PTPRT* transcript levels, as determined by qPCR, in cell models mimicking EGFR hyperactivity (**c**), PTEN deficiency (**d**) or mutant IDH1-mediated 2-HG exposure (**e**). Bars indicate mean with SEM, asterisk represents significance level (* *p* < 0.05)

Strikingly, for 80 out of the 91 PTP genes a pattern emerged showing lower transcript levels in the glioblastoma samples as compared to lower grade gliomas (Fig. 1a-b). This raises the question whether general PTP profiles are influenced by characteristic genetic alterations associated with lower grade glioma (frequently *IDH*-mutant) or glioblastoma (e.g. *EGFR* amplification; *PTEN* deletion) or rather reflect the tumor pathogenesis, including cell of origin. To test this, U-251 MG glioblastoma cells were lentivirally transduced to over-express EGFR or EGFRvIII. Phosphorylation of EGFR and EGFRvIII was readily detected in the transduced cells and was effectively blocked by treatment with the EGFR inhibitor Gefitinib (Fig. 5a). The increased EGFR signaling, however, did not influence expression levels of three representative PTPs (DUSP16, PTPRG and PTPRT; Fig. 5c) that displayed the grade-related expression pattern as observed for the majority of the PTP cohort (Fig. 1a-b).

PTEN-inactivating deletions or mutations represent another common aberration in primary glioblastoma samples and could potentially explain the pattern observed on the PTP transcripts. To genocopy PTEN loss, the PTEN WT glioblastoma cell line LN-229 was subjected to CRISPR/Cas9-mediated genome editing and resulting clones were inspected for PTEN levels and activity, respectively, as witnessed by PTEN and phospho-AKT levels under low-serum conditions (Fig. 5b). Although we successfully generated PTEN-deficient LN-229 derivates and appropriate controls, no significant changes in expression levels were observed for the three representative reporter PTPs that were tested (Fig. 5d).

We also monitored effects of the oncometabolite 2-hydroxyglutarate (2-HG) that is produced in lower grade gliomas and secondary glioblastomas as a result of the characteristic IDH1^R132H^ mutation and inhibits DNA and histone demethylation. Tumor-relevant levels of 2-HG were added for 48 h to U-251 MG (IDH1 wild type) glioblastoma cells and potential changes in DUSP16, PTPRG and PTPRT expression levels were monitored. Addition of 2-HG significantly reduced the mRNA levels for two out of the three PTPs measured (Fig. 5e). These results are in line with epigenetic regulation of *DUSP16* and *PTPRG* but do not explain the generally higher PTP mRNA levels in IDH-mutant low-grade gliomas. RNAseq data from WT or IDH1 R132H-containing glioma xenografts E434 and E478 [27, 33] also do not point to an IDH-mutation associated difference in expression for DUSP16, PTPRG and PTPRT (WPJL, unpublished data). Collectively, these data make it rather unlikely that genetic alterations characterizing lower grade glioma and glioblastoma subgroups are major determinants of the general PTP expression pattern observed (Fig. 1a,b).

### Overexpression of DUSP26 or PTPRT in E98 glioblastoma cells results in reduced tumorigenicity

**Fig. 6:**
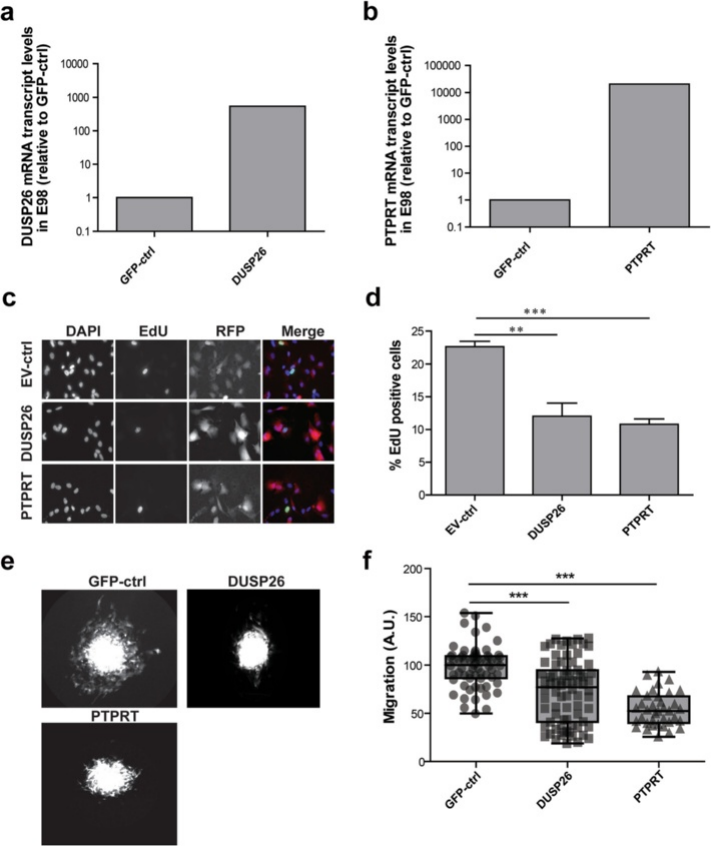
DUSP26 and PTPRT suppress glioblastoma cell growth and motility. DUSP26 (**a**) and PTPRT (**b**) mRNA levels in lentivirally transduced E98 cells were determined by qRT-PCR. Expression, normalized to ACT-B, is given as fold change of that in GFP control cells. **c** Transduced E98 cells were grown on coverslips for 48 h, pulse-labeled with EdU for 1 h, fixed, and EdU incorporation was visualized. Representative images of DAPI, EdU and TagRFP positivity are depicted. **d** Percentage of EdU-positive nuclei among DAPI-stained cells was determined (*n* > 3). Error bars indicate SD and asterisks reflect confidence levels (ANOVA; ** *p* < 0.01; *** *p* < 0.001). **e** Spheroids of transduced E98 cells on a confluent mouse astrocyte layer were incubated for 24 h before being fixed. Representative images of TagRFP-expressing spheroids with outgrowing cells are shown for the respective constructs. **f** The average radius increase (in pixels) due to migrating cells was calculated per spheroid from the data in (**e**), and plotted as gray data points on top of the box plot for the mean with standard deviation. Asterisks indicate significance (*** *p* < 0.001; Kruskal-Wallis one way analysis of variance *p* < 0.0001)

DUSP26 and PTPRT are the most down-regulated PTPs in gliomas with highest grade malignancy and both show a pronounced correlation with survival probability (Figs. 1, 3, 4). To test direct effects of expression on cell behavior we overexpressed these PTPs in the E98 glioblastoma model [11, 21, 27, 34] and monitored effects on cell growth and migration (Fig. 6). To facilitate tracking of successfully transduced E98 cells, lentiviral expression vectors were used that also carry a TagRFP fluorescent reporter. DUSP26 and PTPRT protein expression of virus - producing HEK293FT was visualized using immunoblotting [see Additional file 5]. Successful overexpression of DUSP26 and PTPRT in E98 cells was confirmed by qPCR (Fig. 6a,b) and E98 cell proliferation capacity was investigated using EdU labeling of S-phase cells. Increased DUSP26 or PTPRT levels led to reduced EdU incorporation as compared to empty-vector controls (Fig. 6c-d). This suppression of proliferation in the E98 high grade glioma model following overexpression of these two PTPs is in line with the observed positive correlation with patient survival data.

To also monitor potential DUSP26 or PTPRT effects on glioblastoma cell migration, we turned to a previously established spheroid outgrowth assay [4, 28]. Homogeneously-sized spheroids of lentivirally transduced E98 cells, generated using the hanging-drop method, were placed on a layer of mouse astrocytes and the migratory performance of outgrowing fluorescent E98 cells was calculated as the change in radius of the spheroid over 24 h. As compared to GFP-expressing control cells, the expression of either PTPRT or DUSP26 significantly reduced migration of E98 cells from the spheroid edge (Fig. 6e-f). DUSP26 and PTPRT thus impinge on both growth and motility of glioblastoma cells. Collectively, these data underscore their involvement as tumor suppressors in glioma, in line with the mRNA profiling data (Figs. 1, 3, 4).

## Discussion

Diffuse gliomas are invariably fatal tumors, with most glioblastoma patients surviving only up to 15 months after diagnosis [3], stressing the need for more effective treatment modalities. In this study we focused on the protein tyrosine phosphatase gene family as alternative entry points for treatment strategies. Seven out of ninety-one investigated PTPs displayed significantly different mRNA expression levels when comparing lower and high grade gliomas. Furthermore, for four out of these seven, expression levels correlated with patient survival probability, underscoring their role in tumor behavior. Importantly, overexpression of the two PTPs that showed the largest expression difference in our qPCR and in silico analyses, DUSP26 and PTPRT, resulted in reduced glioblastoma cell proliferation and migration, supportive of a tumor suppressive role.

In general, PTP expression levels in lower grade gliomas resembled those in normal brain tissue whereas levels in glioblastomas were generally lower. This overall reduction in PTP expression with increasing grade may relate to differences in the cell type of origin. Alternatively, it may be imposed by characteristic genetic alterations acquired during glioma development. We found that experimental introduction of EGFR hyperactivity, or of PTEN inactivation, in a glioblastoma cell line had no effect on overall PTP expression patterns. Furthermore, the transcriptional impact of 2-HG on PTP expression in IDH1 wildtype glioblastoma cells was at best opposite to the expected, and not able to tone down PTP mRNA levels to those in lower grade gliomas or normal brain tissue. These findings suggest that other differences in tumor pathogenesis between lower and high grade tumors are the major determinants for the PTP expression pattern. The overall reduced PTP levels in glioblastomas as compared to histologically normal brain and lower grade tumor levels, points to PTP agonists as a potential option for glioblastoma treatment.

In our screening rationale, stringent criteria led to a selection of seven PTPs that differed significantly in their expression between glioblastomas and lower grade gliomas. Our data revealing that *PTEN* expression was low in high grade gliomas concords with literature data on PTEN inactivation or absence in over one-third of high grade gliomas, resulting in increased proliferation and survival through activation of the PI3K/AKT pathway [10, 35]. PTPRM has also been proposed as a glial tumor suppressor (e.g. [19, 32]) and is consistently down-regulated in high grade samples in our screen. Together with its subfamily members PTPRU and PTPRK, they are amongst the most frequently mutated PTPs in a number of cancer types [16, 19, 20, 32, 36]. Some PTPs that had previously been implicated in primary glioblastoma etiology [19] were not selected by our screen, although some only just failed to reach our criteria (*e.g. PTPN11*, encoding SHP2). This may reflect that such PTPs could govern processes equally important to lower grade and high grade tumors.

We found that *PTPRZ1* expression is higher in lower grade tumors than in high grade tumors, but in online RNA-seq data the inverse was observed. This apparent discrepancy may result from histopathological differences in the tumor collections. In the RNA-seq dataset, a third of the lower grade samples represent astrocytomas while in our sample cohort lower grade tumors consisted mostly of oligodendroglial neoplasms with 1p/19q co-deletion. *PTPRZ1* is highly expressed in oligodendrocyte precursor cells and is involved in normal oligodendrocyte development [37, 38], thus providing rationale for the high *PTPRZ1* expression in our sample cohort. Irrespective, *PTPRZ1* expression is consistently up-regulated in all glioma specimens. We and others have shown that the encoded transmembrane PTP acts as an oncogene in gliomas [21, 39, 40] and, interestingly, two HLA-presented peptides derived from PTPRZ1’s extracellular part are included in an experimental glioma vaccine that is currently explored in phase II clinical trials [41].

We additionally identified PTPRT, DUSP26, PTPRN2 and MTMR4 in our screen for glioma-relevant PTPs. PTPRT belongs to the same cell adhesion molecule-like PTP subfamily as PTPRM, PTPRU and PTPRK. Although compelling evidence has put forward *PTPRT* as a colon cancer susceptibility gene [20, 42–44], thus far no reports have linked PTPRT to gliomas. PTPRT mRNA levels are quite low in normal brain tissue and lower grade gliomas but are still on average 40-fold higher than those in high grade glioblastomas. In line with a tumor suppressive role, we found that PTPRT overexpression significantly suppressed glioblastoma cell proliferation and migration. The finding that the closely related PTPRT and PTPRM show distinct expression patterns in lower versus high grade gliomas (PTPRU just failed to reach the criteria in the first cohort) and correlate with patient survival, warrants further studies on their role in glioma-associated signaling pathways.

DUSP26 represents a dual-specificity phosphatase, *i.e*. it is able to dephosphorylate not only phosphotyrosines but also phospho-serine and -threonine residues in substrate proteins. In neuroblastoma cells DUSP26 dephosphorylates and inhibits the tumor suppressor p53 [45]. Furthermore, *DUSP26* amplification has been found in thyroid cancer, and DUSP26 knockdown impeded growth of anaplastic thyroid cancer cells [46]. Collectively, this indicates that *DUSP26* acts as an oncogene, and contrasts with our observation of low DUSP26 levels in glioblastomas in comparison with lower grade glioma samples and normal brain tissue. Importantly, we found that DUSP26 overexpression in E98 glioblastoma cells resulted in decreased growth and motility. Other investigators also reported on down-regulated DUSP26 levels in glioblastoma [47] and Tanuma and colleagues additionally provided evidence that DUSP26 facilitates catenin/cadherin delivery to cell-cell junction sites, hence cell-cell adhesiveness [48]. It is becoming increasingly clear that diffuse glioma cells form functional networks via cell-cell contacts [49] and in such a context DUSP26 protein may indeed have tumor suppressive activity. Together with PTPRM and PTPRT, DUSP26 therefore holds promise for therapeutic intervention.

Intriguingly, *PTPRN2* and *MTMR4* both encode PTPs that, like PTEN, have phosphoinositides as substrates. PTPRN2 preferentially dephosphorylates phosphatidylinositol 4,5-bisphosphate (PI(4,5)P) [50] and MTMR4 prefers phosphatidylinositol 3-phosphate (PI(3)P) [51]. Whereas the tumor suppressive impact of PTEN’s phospholipid phosphatase activity is beyond doubt [52], only limited and contradicting data exist regarding PTPRN2 involvement in cancer. The hypermethylation of the *PTPRN2* promoter region in glioblastomas and lung adenocarcinomas [53, 54] suggests tumor suppressor roles. In contrast, high PTPRN2 expression has been reported to correlate with poor clinical outcomes in breast cancer cases [55]. Furthermore, experimental down-regulation of PTPRN2 in metastatic breast cancer cells inhibited migratory potential and yielded smaller tumors in vivo [56], rather pointing to oncogenic potential. The tumor biological impact of PTPRN2 and MTMR4 may thus be context-dependent, and how these PTPs feed into glioma biology needs further investigation.

## Conclusions

Taken together, our study – to our knowledge the first comprehensive PTP mRNA profiling in diffuse gliomas – highlights seven PTPs (DUSP26, MTMR4, PTEN, PTPRM, PTPRN2, PTPRT and PTPRZ1) of which expression decreases with increasing malignancy. Largest expression differences between lower grade and high grade diffuse gliomas were found for DUSP26 and PTPRT. Their expression correlated with patient survival probability, and overexpression inhibited glioblastoma cell growth and motility. Together with PTPs that impinge upon cellular contacts (DUSP26, PTPRZ1) and phospholipid signaling (MTMR4, PTEN and PTPRN2) they provide novel cues to explore and design glioma treatment options.

## Additional files

Additional file 1: Molecular pathological characteristics of glioma samples used in this study. (PDF 309 kb) Additional file 2: ΔΔCt values of 1st cohort of diffuse glioma samples run for 91 validated primer sets. (PDF 490 kb) Additional file 3: ΔΔCt values of 2nd cohort of diffuse glioma samples run for 36 validated primer sets judged as candidates in the 1st cohort. (PDF 470 kb) Additional file 4: Immunohistochemical staining for PTPRT on formalin-fixed paraffin-embedded materials. (PDF 303 kb) Additional file 5: HEK293FT cells expressing lentiviral constructs for DUSP26 and PTPRT. (PDF 192 kb)
