# Supplementary material for: Comprehensive protein tyrosine phosphatase mRNA profiling identifies new regulators in the progression of glioma
Source: Acta Neuropathol Commun. 2016 Sep 1;4(1):96. doi: 10.1186/s40478-016-0372-x (PMC5009684; doi:10.1186/s40478-016-0372-x)
Supplement: Additional file 1: — Molecular pathological characteristics of glioma samples used in this study. (PDF 309 kb) [file 40478_2016_372_MOESM1_ESM.pdf]

Additional file 1 : Molecular pathological characteristics of glioma samples used in this study.

# sample	grade	1p	19q	IDH1 mut	EGFR	EGFRvIII	PTEN
1	II	loss	loss	Yes	normal	normal	normal
2	II	loss	loss	Yes	normal	normal	normal
3	II	normal	normal	Yes	normal	normal	normal
4	II	ND	ND	Yes	ND	ND	ND
5	II	loss	loss	No	normal	normal	normal
6	II	loss	loss	Yes	normal	normal	normal
7	II	loss	loss	Yes	normal	normal	normal
8	II	loss	loss	Yes	normal	normal	normal
9	II	loss	partial gain	Yes	normal	normal	gain
10	II	loss	loss	No	normal	normal	normal
11	II	loss	loss	Yes	normal	normal	normal
12	II	loss	loss	ND	ND	ND	ND
13	II	loss	loss	ND	ND	ND	ND
14	II	ND	ND	Yes	ND	ND	ND
15	II	normal	normal	ND	normal	normal	normal
16	II	loss	loss	Yes	normal	normal	normal
17	II	loss	loss	Yes	normal	normal	normal
18	II	ND	ND	ND	ND	ND	ND
19	II	loss	loss	Yes	normal	normal	normal
20	III	normal	normal	Yes	normal	normal	loss
21	III	loss	loss	Yes	normal	normal	normal
22	III	loss	loss	Yes	normal	normal	normal
23	III	loss	loss	Yes	normal	normal	normal
24	III	ND	ND	Yes	ND	ND	ND
25	III	ND	ND	Yes	ND	ND	ND
26	III	normal	normal	ND	HCA	variant	loss
27	III	ND	ND	Yes	ND	ND	ND
28	III	loss	loss	Yes	normal	normal	normal
29	III	loss	loss	Yes	HCA	normal	normal
30	III	ND	ND	No	gain	normal	normal
31	III	ND	ND	Yes	normal	normal	loss
32	III	loss	loss	Yes	normal	normal	normal
33	III	loss	loss	Yes	normal	normal	normal
34	III	loss	loss	Yes	normal	normal	normal
35	IV	normal	normal	No	normal	normal	loss
36	IV	normal	normal	No	gain	normal	loss
37	IV	normal	normal	No	gain	normal	loss
38	IV	partial loss 1p36	loss	No	HCA	variant	loss
39	IV	normal	partial gain	No	HCA	variant	loss
40	IV	normal	normal	No	gain	normal	normal

**41**	IV	partial loss	normal	No	HCA	variant	loss
**42**	IV	loss	partial loss	No	HCA	variant	loss
**43**	IV	ND	ND	No	ND	ND	ND
**44**	IV	loss	partial loss	No	HCA	normal	loss
**45**	IV	partial loss	partial gain	No	HCA	normal	HD
**46**	IV	normal	gain	No	HCA	normal	normal
**47**	IV	partial loss	partial gain/partial loss	No	HCA	other	normal
**48**	IV	loss	normal	No	HCA	variant	loss
**49**	IV	ND	ND	No	HCA	normal	normal
**50**	IV	ND	ND	No	ND	ND	ND
**51**	IV	ND	ND	No	ND	ND	ND
**52**	IV	loss	gain	No	HCA	variant	loss
**53**	IV	ND	ND	No	gain	normal	loss
**54**	IV	ND	ND	No	gain	normal	loss
**55**	IV	ND	ND	No	normal	normal	normal
**56**	IV	ND	ND	No	ND	ND	ND
**57**	IV	ND	ND	No	gain	normal	loss
**58**	IV	ND	ND	No	normal	normal	normal
**59**	IV	ND	ND	No	HCA	variant	loss
**60**	IV	normal	gain	No	HCA	normal	loss
**61**	IV	normal	gain	No	HCA	normal	loss
**62**	IV	ND	ND	No	HCA	normal	normal
**63**	IV	normal	partial loss 19pter-q24	No	normal	normal	normal
**64**	IV	normal	normal	No	ND	ND	ND
**65**	IV	normal	normal	No	HCA	variant	HD
**66**	IV	ND	ND	No	ND	ND	ND
**67**	IV	ND	ND	No	ND	ND	ND
**68**	IV	normal	normal	No	normal	normal	normal
**69**	IV	normal	partial gain	No	gain	normal	loss
**70**	IV	normal	normal	No	HCA	variant	normal
**71**	IV	normal	normal	No	gain	normal	loss
**72**	IV	normal	normal	No	gain	normal	loss
**73**	IV	partial gain	partial gain	No	normal	normal	normal
**74**	IV	normal	normal	Yes	normal	normal	normal
**75**	IV	normal	normal	No	gain	normal	normal
**76**	IV	normal	normal	No	HCA	normal	normal
**77**	IV	normal	normal	No	gain	normal	loss
**78**	IV	normal	normal	No	HCA	variant	normal
**79**	IV	normal	gain	Yes	normal	normal	normal
**80**	IV	normal	normal	No	normal	normal	loss
**81**	IV	ND	ND	No	normal	normal	normal
**82**	IV	ND	ND	No	ND	ND	ND
**83**	IV	partial gain 4-14	partial loss 19-21	No	normal	normal	normal

MLPA (Multiplex Ligation-dependent Probe Amplification)* was used to detect 1p19q, EGFR, EGFRvIII and PTEN status, and IDH status was reviewed using R132H-specific immunohistochemical staining and DNA sequencing by the diagnostic department of the Radboudumc. ND, not determined; HCA, high copy amplification; HD, homozygous deletion.

* Jeuken J, Cornelissen S, Boots-Sprenger S, Gijsen S, Wesseling P: **Multiplex ligation-dependent probe amplification: a diagnostic tool for simultaneous identification of different genetic markers in glial tumors.** *J Mol Diagn.* 2006, **8**:433-443.

Comprehensive protein tyrosine phosphatase mRNA profiling identifies new regulators in the progression of glioma

Acta Neuropathologica Communications

Bourgonje, Verrijp, Schepens, Navis, Piepers, Palmen, van den Eijnden, Hooft van Huijsduijnen, Wesseling, Leenders and Hendriks
