# Supplementary material for: Comprehensive protein tyrosine phosphatase mRNA profiling identifies new regulators in the progression of glioma
Source: Acta Neuropathol Commun. 2016 Sep 1;4(1):96. doi: 10.1186/s40478-016-0372-x (PMC5009684; doi:10.1186/s40478-016-0372-x)
Supplement: Additional file 2: — ΔΔCt values of 1st cohort of diffuse glioma samples run for 91 validated primer sets. (PDF 490 kb) [file 40478_2016_372_MOESM2_ESM.pdf]

Additional file 2: ΔΔCt values of 1st cohort of diffuse glioma samples run for 91 validated primer sets.

	1st cohort								
	Lower grade (II-III)			*glioblastoma (IV)*			*lower grade vs glioblastoma*		*Meets*
	avg	*sem*	*n*	*avg*	*sem*	*n*	*p-value*	*diff*	*criteria*
**ACP1**	-3.25155	0.557863	14	-4.39231	0.318133	18	0.0708	1.140767	
**CDC14A**	-1.97436	0.525319	13	-3.21579	0.232678	19	0.022413	1.24143	
**CDC14B**	-1.21846	0.356338	13	-2.00965	0.298728	19	0.100029	0.791188	
**CDC25A**	-3.74155	0.474555	14	-4.13338	0.311585	20	0.476282	0.391827	
**CDC25B**	1.379778	0.408468	15	0.923728	0.353846	19	0.40357	0.45605	
**CDC25C**	-9.52429	0.682148	14	-9.82289	0.290424	19	0.660844	0.298609	
**CDKN3**	2.389936	0.749589	13	1.151574	0.507078	18	0.166087	1.238362	
**DUSP01**	0.556218	0.34075	13	0.585046	0.328256	18	0.952823	0.028828	
**DUSP02**	-2.98639	0.862621	12	-4.4164	0.423927	19	0.10918	1.430015	
**DUSP03**	-1.30583	0.214426	12	-2.33185	0.200181	18	0.002029	1.026019	
**DUSP04**	-5.37845	0.756125	14	-6.00535	0.332932	19	0.41193	0.626898	
**DUSP05**	-7.65548	0.524687	14	-8.2555	0.507834	20	0.42939	0.600024	
**DUSP06**	2.755972	0.804182	12	-0.02509	0.356631	18	0.001413	2.781065	*
**DUSP07**	-0.85692	0.354814	13	-2.68548	0.211657	19	5.34E-05	1.828559	*
**DUSP08**	-1.28789	0.657521	15	-0.75222	0.8871	21	0.655473	0.535667	
**DUSP09**	-1.97403	0.721805	12	-4.23675	0.688777	21	0.041527	2.262718	*
**DUSP10**	-1.11405	0.490657	14	-1.25037	0.445964	18	0.83911	0.136323	
**DUSP11**	2.601515	0.619086	11	0.71912	0.200357	18	0.001793	1.882395	*
**DUSP12**	3.363077	0.59329	13	1.331272	0.241018	19	0.001208	2.031805	*
**DUSP14**	0.100385	0.595505	13	-0.38833	0.316979	18	0.442054	0.488718	
**DUSP16**	0.42	0.371427	12	-0.95948	0.232409	16	0.002816	1.379479	
**DUSP18**	-7.36107	0.630275	14	-7.02454	0.680874	18	0.72621	0.336534	
**DUSP19**	-1.73538	0.334111	13	-3.36325	0.285807	20	0.000944	1.627865	*
**DUSP22**	-0.54744	0.252797	13	-1.092	0.360588	20	0.277898	0.544564	
**DUSP23**	-0.37472	0.603368	12	-0.42046	0.228557	18	0.935895	0.045741	
**DUSP26**	-0.03712	0.4471	13	-2.90769	0.497295	18	0.000298	2.87057	*
**EPM2A**	-0.56929	0.684909	13	-2.17883	0.943768	20	0.224354	1.609538	
**MTM01**	-0.0859	0.322624	13	-2.01318	0.259416	16	6.59E-05	1.92728	*
**MTMR02**	0.261154	0.362867	13	-0.17798	0.425436	19	0.467465	0.439136	
**MTMR03**	-0.79205	0.253864	13	-1.23658	0.40893	20	0.424227	0.444532	
**MTMR04**	0.199487	0.338745	13	-1.93224	0.378075	19	0.000415	2.131724	*
**MTMR06**	-0.56295	0.324275	13	-1.86691	0.242742	17	0.002719	1.303963	
**MTMR07**	-2.23744	0.656055	13	-5.07514	0.672648	18	0.006574	2.837703	*
**MTMR08**	-0.62607	0.772293	14	-1.16489	0.759163	15	0.623044	0.538817	
**MTMR09**	-0.4891	0.335848	13	-1.79842	0.208855	19	0.0015	1.309318	
**MTMR10**	-1.09679	0.443789	13	-3.19627	0.5258	19	0.007746	2.099477	*
**MTMR11**	-2.90958	0.688153	12	-0.33809	1.935293	17	0.290677	2.571495	

**MTMR12**	-0.90917	0.235381	12	-2.69671	0.277676	18	9.09E-05	1.787546	*
**PTEN**	0.768889	0.499919	12	-1.66537	0.277297	18	8.33E-05	2.434259	*
**PTP4A1**	3.006474	0.810708	13	-0.19241	0.273705	18	0.000216	3.198882	*
**PTP4A2**	-0.78353	0.373092	13	-2.11523	0.335218	18	0.013505	1.331706	
**PTP4A3**	-0.21481	0.236823	13	-0.84301	0.461602	18	0.288683	0.628202	
**PTPDC1**	-2.08103	0.286822	13	-2.16983	0.379175	20	0.866917	0.088808	
**PTPMT1**	0.092692	0.344434	13	-2.12579	0.525013	19	0.003419	2.218482	*
**PTPN01**	-1.12356	0.817924	12	-1.57782	0.678055	18	0.673202	0.454262	
**PTPN02**	-1.49834	0.722416	12	-1.41565	0.360869	18	0.911153	0.082694	
**PTPN03**	-4.25103	0.513095	15	-5.42329	0.521144	20	0.126875	1.172266	
**PTPN04**	0.014509	0.249476	15	-2.0019	0.263667	21	6.04E-06	2.016413	*
**PTPN05**	-3.60516	0.642601	15	-6.47111	0.664014	21	0.005054	2.865948	*
**PTPN06**	0.803166	0.360692	12	0.174028	0.429042	18	0.30635	0.629138	
**PTPN07**	-0.33067	0.692397	15	1.00307	0.517463	19	0.124999	1.333742	
**PTPN09**	0.692678	0.144811	15	-0.95554	0.532223	20	0.013224	1.64822	*
**PTPN11**	1.474958	0.61778	12	-1.29421	0.409221	19	0.000528	2.769169	*
**PTPN12**	1.07932	0.182015	15	1.142125	0.485565	20	0.915045	0.062805	
**PTPN13**	0.075712	0.237878	14	-0.83688	0.371977	20	0.070904	0.912587	
**PTPN14**	-0.47522	0.453636	15	-1.04804	0.378208	20	0.336503	0.572819	
**PTPN18**	0.651222	0.608698	15	0.062083	0.515059	18	0.462405	0.589139	
**PTPN21**	-0.38167	0.298817	14	-0.53204	0.21877	20	0.680202	0.150375	
**PTPN22**	-1.56313	0.611956	12	-1.53804	0.588186	17	0.977191	0.025086	
**PTPN23**	0.159583	0.685866	12	-1.62132	0.240633	19	0.007511	1.780899	*
**PTPRA**	-0.46128	0.341797	15	-1.3556	0.208479	21	0.024257	0.894317	
**PTPRB**	0.550536	0.31794	14	-1.29079	0.264417	20	9.54E-05	1.841327	*
**PTPRC**	0.971488	0.542533	14	0.644405	0.377564	21	0.612553	0.327083	
**PTPRD**	1.396488	0.254753	14	-0.65576	0.270189	18	7.58E-06	2.052252	*
**PTPRE**	1.025893	0.511706	14	-1.16558	0.247028	21	0.000159	2.191468	*
**PTPRF**	-0.39993	0.299968	12	-1.3413	0.345972	18	0.065208	0.941366	
**PTPRG**	0.375495	0.298564	13	-0.0102	0.546258	21	0.603925	0.385694	
**PTPRH**	-3.6776	0.782694	13	-2.76467	0.503051	20	0.310795	0.912936	
**PTPRJ**	0.449366	0.369573	13	-1.29037	0.358437	18	0.002528	1.739736	*
**PTPRK**	0.435372	0.508261	14	-0.27196	0.315817	20	0.221788	0.707331	
**PTPRM**	-1.24503	0.333427	16	-2.8186	0.244773	22	0.000406	1.573564	*
**PTPRN**	-1.26161	0.812509	14	-3.52429	0.631556	20	0.033011	2.262683	*
**PTPRN2**	-0.27096	0.35539	15	-2.91737	0.383316	20	2.44E-05	2.646413	*
**PTPRO**	-1.40913	0.48252	13	-3.4707	0.412299	19	0.003014	2.061574	*
**PTPRQ**	-3.59416	0.701952	12	-2.34721	0.65876	20	0.226981	1.246948	
**PTPRR**	-2.34379	0.420771	12	-2.82475	0.383227	20	0.423885	0.480958	
**PTPRS**	0.609453	0.287494	14	-1.04475	0.297355	20	0.000532	1.654203	*
**PTPRT**	-0.18173	0.558499	13	-5.37245	0.681148	18	5.32E-06	5.190727	*
**PTPRU**	-1.70976	0.525682	15	-3.11982	0.401263	19	0.037412	1.410069	
**PTPRZ1**	3.064411	0.22548	12	1.164667	0.415288	20	0.002179	1.899744	*

**RNGTT**	-0.71197	0.43516	12	-2.55412	0.1851	18	0.000146	1.842151	*
**SBF1**	-4.17682	0.334524	11	-4.873	0.295629	20	0.149577	0.696182	
**SBF2**	0.592087	0.416762	12	-1.31384	0.196047	18	8.39E-05	1.90593	*
**SSH1**	-0.26894	0.303309	12	-1.09356	0.159327	18	0.013914	0.824626	
**SSH2**	0.33569	0.436566	13	-1.46745	0.209653	18	0.00034	1.803143	*
**SSH3**	-2.29922	0.471247	12	-0.75587	0.397372	21	0.021172	1.543347	*
**STYX**	0.095482	0.510495	13	-1.5102	0.315042	17	0.009073	1.605678	*
**STYXL1**	0.109339	0.375299	12	-0.44214	0.409861	21	0.375752	0.551482	
**TENC1**	-2.42665	0.331074	13	-2.79392	0.408297	20	0.526917	0.367266	
**TNS1**	-3.5168	0.326485	13	-4.57775	0.346491	20	0.043818	1.060949	
**TNS3**	-0.71594	0.42453	12	-1.8937	0.223498	18	0.012332	1.177765	

The relative expression of PTPs compared to histologically normal brain tumor in different malignancy grades lower (grade II-III) vs high grade (grade IV) diffuse gliomas. PTPs are arranged alphabetically. *Avg (* average ΔΔ C_t across samples for a single PTP). *sem* (standard error of the mean of the samples in avg, *n* (number of samples tested in the first cohort per group). This varies per PTP because expression of some PTPs is so low that only a high amount of RNA and cDNA will show a reliable $\Delta\Delta C_t$ value. *P-value* (statistically tested using Student t-test, calculated between lower-grade and high-grade glioma samples from the previous columns). *diff* (absolute difference in $\Delta\Delta C_t$ between lower grade and high grade glioma samples). A candidate is defined as $p<0.05$ and $|\Delta\Delta C_t| > 1.5$.

Comprehensive protein tyrosine phosphatase mRNA profiling identifies new regulators in the progression of glioma

Acta Neuropathologica Communications

Bourgonje, Verrijp, Schepens, Navis, Piepers, Palmen, van den Eijnden, Hooft van Huijsduijnen, Wesseling, Leenders and Hendriks
