# Supplementary material for: Comprehensive protein tyrosine phosphatase mRNA profiling identifies new regulators in the progression of glioma
Source: Acta Neuropathol Commun. 2016 Sep 1;4(1):96. doi: 10.1186/s40478-016-0372-x (PMC5009684; doi:10.1186/s40478-016-0372-x)
Supplement: Additional file 3: — ΔΔCt values of 2nd cohort of diffuse glioma samples run for 36 validated primer sets judged as candidates in the 1st cohort. (PDF 470 kb) [file 40478_2016_372_MOESM3_ESM.pdf]

Additional file 3: ΔΔCt values of 2nd cohort of diffuse glioma samples run for 36 validated primer sets judged as candidates in the 1st cohort.

	2nd cohort								
	Lower grade(II-III)			*glioblastoma (IV)*			*Lower grade vs glioblastoma*		*Meets*
	avg	*sem*	*n*	*avg*	*sem*	*n*	*p-value*	*diff*	*criteria*
**DUSP06**	2.856667	0.745139	14	1.805132	0.379352	19	0.184901	1.051535	
**DUSP07**	-1.5205	0.225323	15	-2.06682	0.303028	16	0.162969	0.546323	
**DUSP09**	0.047143	0.70536	14	-1.56267	0.745825	15	0.129664	1.60981	
**DUSP11**	0.291369	0.867016	14	0.799389	0.302442	15	0.574262	0.50802	
**DUSP12**	2.259679	0.73258	13	1.908194	0.366295	17	0.64878	0.351486	
**DUSP19**	-0.14333	0.520413	12	-1.60921	0.361781	16	0.024477	1.465873	
**DUSP26**	2.110476	0.642162	14	-0.51627	0.684359	16	0.009745	2.62675	*
**MTM01**	-1.97095	0.596229	14	-0.70724	0.353405	16	0.070845	1.263713	
**MTMR04**	-0.83115	0.38667	13	-2.47217	0.582182	15	0.031626	1.641013	*
**MTMR07**	-2.06826	0.725815	12	-3.12036	0.581401	16	0.262851	1.052101	
**MTMR10**	-2.23991	0.435974	14	-2.25299	0.352138	17	0.981319	0.013083	
**MTMR12**	-2.52821	0.403529	13	-1.92574	0.344054	18	0.26547	0.602464	
**PTEN**	3.210539	0.737889	17	0.695708	0.494792	20	0.006336	2.514831	*
**PTP4A1**	3.113462	0.778614	13	2.745602	0.364403	18	0.643235	0.36786	
**PTPMT1**	-0.85323	0.505893	16	-1.75818	0.729131	16	0.316014	0.904948	
**PTPN04**	-0.13468	0.398514	14	-0.00946	0.397168	20	0.830616	0.125224	
**PTPN05**	-2.5195	0.644852	12	-3.65596	0.763076	20	0.313182	1.136462	
**PTPN09**	2.52591	0.525052	14	1.414042	0.300955	20	0.058452	1.111869	
**PTPN11**	1.157402	0.782	13	-0.11224	0.246148	19	0.083074	1.269639	
**PTPN23**	2.092238	0.735329	15	1.29297	0.790034	17	0.468531	0.799268	
**PTPRB**	-0.09375	0.40724	12	-0.10176	0.567361	18	0.991804	0.008009	
**PTPRD**	0.815	0.424222	16	-0.02468	0.328476	18	0.12312	0.839676	
**PTPRE**	1.91	0.397673	12	0.980219	0.44406	19	0.159192	0.929781	
**PTPRJ**	-0.0585	0.412693	13	0.552807	0.467766	19	0.36297	0.611303	
**PTPRM**	0.241506	0.318901	15	-1.35033	0.630703	15	0.032326	1.591839	*
**PTPRN**	-0.30016	0.501415	13	-1.51421	0.622846	18	0.163595	1.214054	
**PTPRN2**	0.950112	0.521216	15	-0.98242	0.586004	20	0.023605	1.932529	*
**PTPRO**	-0.13803	0.623377	15	-1.74478	0.477837	15	0.050289	1.606745	
**PTPRS**	0.297587	0.370615	13	-0.76538	0.378555	20	0.065821	1.062962	
**PTPRT**	2.566557	0.990977	16	-3.89536	0.613803	16	5.02E-06	6.461921	*
**PTPRZ1**	4.233353	0.560277	14	0.890044	0.432969	19	3.81E-05	3.343309	*
**RNGTT**	-1.48855	0.355342	16	-1.65495	0.210277	18	0.681791	0.1664	
**SBF2**	-0.12773	0.404277	15	-0.73711	0.205654	17	0.174211	0.609375	
**SSH2**	0.062055	0.614237	13	-0.75294	0.3962	15	0.262849	0.815	
**SSH3**	1.368139	0.582929	12	2.801444	0.545805	15	0.086337	1.433306	
**STYX**	0.321298	0.60187	14	-0.25267	0.67783	15	0.534154	0.573965	

The relative expression of PTPs compared to histologically normal brain tumor in different malignancy grades lower (grade II-III) vs high grade (grade IV) diffuse gliomas. PTPs are arranged alphabetically. *Avg (* average $\Delta\Delta C_t$ across samples for a single PTP). *sem* (standard error of the mean of the samples in avg, *n* (number of samples tested in the second cohort per group. *P-value* (statistically tested using Student *t*-test, calculated between lower grade and high grade glioma samples from the previous columns). *diff* (absolute difference in $\Delta\Delta C_t$ between lower-grade and high-grade glioma samples). A candidate is defined as $p<0.05$ and $|\Delta\Delta C_t|> 1.5$.

Comprehensive protein tyrosine phosphatase mRNA profiling identifies new regulators in the progression of glioma

Acta Neuropathologica Communications

Bourgonje, Verrijp, Schepens, Navis, Piepers, Palmen, van den Eijnden, Hooft van Huijsduijnen, Wesseling, Leenders and Hendriks
