# Supplementary material for: Comprehensive protein tyrosine phosphatase mRNA profiling identifies new regulators in the progression of glioma
Source: Acta Neuropathol Commun. 2016 Sep 1;4(1):96. doi: 10.1186/s40478-016-0372-x (PMC5009684; doi:10.1186/s40478-016-0372-x)
Supplement: Additional file 4: — Immunohistochemical staining for PTPRT on formalin-fixed paraffin-embedded materials. (PDF 303 kb) [file 40478_2016_372_MOESM4_ESM.pdf]

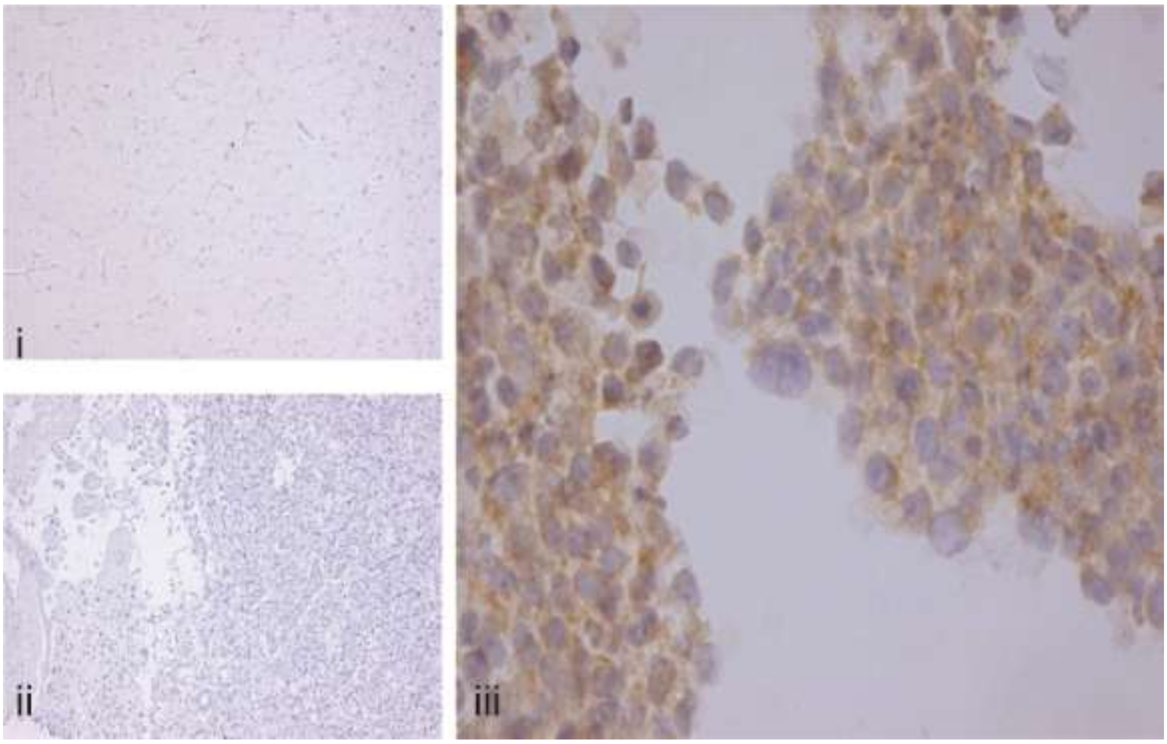

Additional file 4

Immunohistochemical staining for PTPRT on formalin-fixed paraffin-embedded materials. Three different samples stained with antiserum against PTPRT: **i**) histologically normal brain tissue; **ii**) glioma sample; **iii**) HEK293FT cells transfected with a PTPRT expression construct.

Comprehensive protein tyrosine phosphatase mRNA profiling identifies new regulators in the progression of glioma

Acta Neuropathologica Communications

Bourgonje, Verrijp, Schepens, Navis, Piepers, Palmen, van den Eijnden, Hooft van Huijsduijnen, Wesseling, Leenders and Hendriks
