# Supplementary material for: Comprehensive protein tyrosine phosphatase mRNA profiling identifies new regulators in the progression of glioma
Source: Acta Neuropathol Commun. 2016 Sep 1;4(1):96. doi: 10.1186/s40478-016-0372-x (PMC5009684; doi:10.1186/s40478-016-0372-x)
Supplement: Additional file 5: — HEK293FT cells expressing lentiviral constructs for DUSP26 and PTPRT. (PDF 192 kb) [file 40478_2016_372_MOESM5_ESM.pdf]

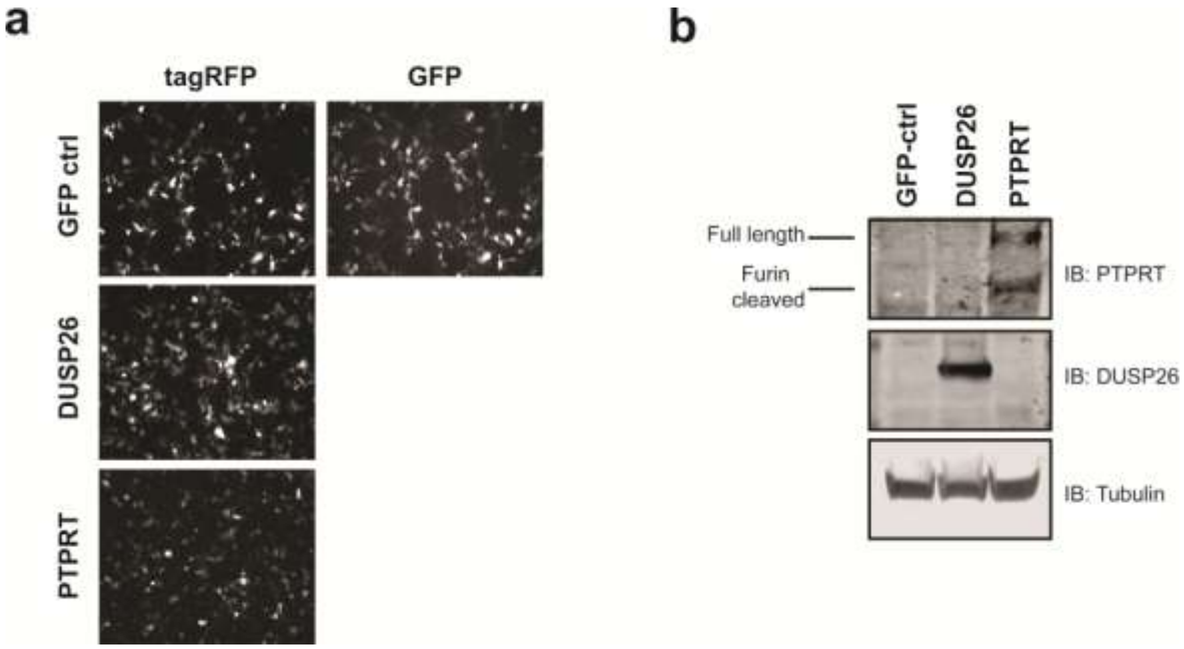

Additional file 5

HEK293FT cells expressing lentiviral constructs for DUSP26 and PTPRT. **a**) Fluorescent images of HEK293FT cells expressing DUSP26, PTPRT, or GFP as a control, under a PGK promoter. Expression constructs also contained a CMV promoter-driven cassette for tagRFP, allowing assessment of transfection efficiency. **b**) Immunoblot of lysates from HEK293FT cells transfected with lentiviral expression constructs for DUSP26, PTPRT or GFP control, stained with antisera against DUSP26, PTPRT and Tubulin.

Comprehensive protein tyrosine phosphatase mRNA profiling identifies new regulators in the progression of glioma

Acta Neuropathologica Communications

Bourgonje, Verrijp, Schepens, Navis, Piepers, Palmen, van den Eijnden, Hooft van Huijsduijnen, Wesseling, Leenders and Hendriks
